# Doctors versus Drink

**Published:** 1895-12-07

**Authors:** 


### Doctors versus Drink.

The census returns for 1891, which have been recently published, disclose the fact that during the last quarter of a century in England and Wales there has been an enormous increase in the number of medical practitioners. So great was the increase in the decade 1881-1891, that it amounted to no less than 25 per cent. In the same period there was an equally remarkable decrease in the number of wine merchants, beersellers, and other persons engaged in the sale of intoxicating liquors. The total number of medical practitioners in England and Wales in 1891 was 18,936. The total number of wine and spirit merchants in 1891 was 7,395; whereas the number in 1871 was 11,217. The number of beersellers in 1891 was 11,816, as against a total of 13,789 in 1871. These returns are relatively incomplete, inasmuch as they obviously take no account of the regular hotelkeepers, who are, of course, a far more numerous class than either beersellers or wine and spirit merchants. Nevertheless, they afford a general indication of the set of the current towards a wider diffusion of medical knowledge, and a corresponding diminution in the quantity of alcoholic liquor consumed in the two periods.

It is affirmed that the diminution in the consumption of alcoholic liquors in the proportions indicated by these figures is an advantage to the community which cannot be measured by any of the ordinary terms of measurement. It is claimed that for this immeasurable advantage the country is largely indebted to the science and generous self-denial of the medical profession. The medical profession has gradually made the fact clear to itself that alcohol, except in extremely limited quantities, is a slow but deadly poison to the human organism. It is a poison which produces illness of a chronic character; and illness of a chronic character puts much money into the doctor's pocket. And yet the doctor, individually, and as a profession, has lifted up his voice against the abuse of alcohol with all the attention-compelling vehemence of an ancient Hebrew prophet. "The doctor would have been a monster if he had done otherwise," the sceptic is ready to exclaim. If that be so, then there are a great many monsters living, thriving, and receiving honour among us. The great brewer knows that hundreds of workmen are injured and ruined by the abuse of his beer. Does he ever try by any means whatever to limit the production of his beer or to warn men against the excessive use of it? As a matter of fact, he sells all he can, and opens new houses wherever he can obtain them, to increase by all means the consumption of his wares.

Now is it true, or is it not, that the doctor acts from any different motives here from those which influence the producers and distributors of alcoholic liquors? If it be said that it would be ridiculous to ask a manufacturer or merchant to act upon any other principles than those which guide ordinary men in the conduct of their business, merely because he happens to manufacture or sell strong drink, then, we ask, why should it not also be thought ridiculous to ask the doctor to behave in a way to the public which is calculated seriously to diminish his own profits—that is, to ask him to act on principles the exact opposite of those acted upon by the ordinary manufacturer or merchant?

We insist, and our contention will bear the severest critical examination, that the doctor has here reached an elevation of principle and conduct which is quite above that of the ordinary man; and we claim for the profession as a whole the recognition of this honourable fact. The voice of science in the mouth of the doctor does not utter abstract propositions, or publish facts of mere academic interest. He is thoroughly in earnest in all his science, and in the application of his science to public and bedside work; and it is for the public to recognise his intelligence and earnestness; and to show its appreciation of both by respecting, learning, and practising all that the doctor has to teach.

